# Detection of dietetically absorbed maize-derived microRNAs in pigs

**DOI:** 10.1038/s41598-017-00488-y

**Published:** 2017-04-05

**Authors:** Yi Luo, Pengjun Wang, Xun Wang, Yuhao Wang, Zhiping Mu, Qingzhi Li, Yuhua Fu, Juan Xiao, Guojun Li, Yao Ma, Yiren Gu, Long Jin, Jideng Ma, Qianzi Tang, Anan Jiang, Xuewei Li, Mingzhou Li

**Affiliations:** 1 Institute of Animal Genetics and Breeding, College of Animal Science and Technology, Sichuan Agricultural University, Chengdu, 611130 China; 2 Chongqing Three Gorges University, Chongqing, 404000 China; 3 The Fishery Institute of Sichuan Academy of Agricultural Sciences, Chengdu, 611731 China; 4 College of Animal Science and Technology, Huazhong Agricultural University, Wuhan, 430070 China; 5 Animal Breeding and Genetics Key Laboratory of Sichuan Province, Sichuan Animal Science Academy, Chengdu, 610066 China

## Abstract

MicroRNAs are a class of small RNAs that are important in post-transcriptional gene regulation in animals and plants. These single-stranded molecules are widely distributed in organisms and influence fundamental biological processes. Interestingly, recent studies have reported that diet-derived plant miRNAs could regulate mammalian gene expression, and these studies have broadened our view of cross-kingdom communication. In the present study, we evaluated miRNA levels in cooked maize-containing chow diets, and found that plant miRNAs were resistant to the harsh cooking conditions to a certain extent. After feeding fresh maize to pigs (7 days), maize-derived miRNAs could be detected in porcine tissues and serum, and the authenticity of these plant miRNAs was confirmed by using oxidization reactions. Furthermore, *in vivo* and *in vitro* experiments demonstrated that dietary maize miRNAs could cross the gastrointestinal tract and enter the porcine bloodstream. In the porcine cells, we found that plant miRNAs are very likely to specifically target their endogenous porcine mRNAs and influence gene expression in a fashion similar to that of mammalian miRNAs. Our results indicate that maize-derived miRNAs can cross the gastrointestinal tract and present in pigs, and these exogenous miRNAs have the potential to regulate mammalian gene expression.

## Introduction

MicroRNAs (miRNAs) are a class of small (18–24 nt) non-coding RNAs that play a critical role in regulating gene expression in animals and plants by binding to target gene transcripts to inhibit their translation or degrade them^1, 2^. These single-stranded molecules are evolutionarily conserved among many species, and influence fundamental biological processes including cell proliferation, differentiation, apoptosis, immune responses, and metabolism^1, 3–7^. miRNAs are widely distributed in mammalian tissues, and their aberrant expression has been associated with numerous diseases^6, 8, 9^. Recently, it has been widely reported that miRNAs can be packed into exosomes (microvesicles) to resist harsh conditions (e.g., RNase and extreme pH) and transferred into neighboring or distant cells to regulate cell function^10, 11^. Stable miRNAs can be detected in nearly all bodily fluids, including serum, milk, saliva and urine^12–15^. Circulating miRNAs are now emerging as a new group of messengers and effectors in intercellular communication, and some of the unique expression patterns of miRNAs reflect various physiological and pathological conditions^16, 17^.

miRNAs show a high degree of conservation in their sequences and mechanisms of action among different organisms^1^. Thus, it is possible for miRNAs to mediate cross-kingdom communication. In 2011, Zhang *et al.* first reported that plant-derived miRNAs could cross the mammalian gastrointestinal (GI) tract into the serum, and demonstrated that plant miR-168a could bind to the mRNA of low-density lipoprotein receptor adapter protein 1 (*LDLRAP1*), inhibit the expression of this protein in the liver and decrease the removal of low-density lipoprotein^18^. Although there is controversy about cross-kingdom regulation by plant miRNAs^19–23^, subsequent studies strongly support the view that exogenous plant miRNAs, as inter-species mediators, are involved in cross-kingdom regulation^24–30^. For example, honeysuckle-derived miR2911 inhibited influenza A viruses^24^, oral administration of three mammalian miRNAs that were 2′-O-methylated like plant miRNAs reduced the intestinal tumor burden^26^ and plant miR-159 inhibited breast cancer growth across kingdoms^25^.

Pig (*Sus scrofa*), an important livestock species, is emerging as an attractive biomedical model due to it having metabolic features, cardiovascular systems and proportional organ sizes similar to those of human^31–33^. In the modern pig industry, commercial pigs are supplied with starch-rich concentrates mainly consisting of maize (as much as 40% or more)^34^. In this context, we performed *in vivo* and *in vitro* experiments in pigs to clarify whether plant-derived miRNAs can be absorbed by mammals and mediate cross-kingdom communication.

## Results

### Identification of abundant miRNAs in fresh maize and chow diets

We used small RNA-seq to reveal the miRNA abundance in fresh maize and observed that the majority of miRNAs with abundant counts were represented by a few miRNAs. The miRNA transcriptome of fresh maize consisted of unevenly distributed sequence counts, among which the top seven miRNAs with the highest expression levels accounted for 85.53% (by reads) of the total reads of all 124 miRNAs (Supplementary Table S1). The abundance of 18 representative miRNAs (10 miRNAs from 80,000–4,000 reads; 2 miRNAs from 2,000–1,000 reads; 2 miRNAs from 400–100 reads; 2 miRNAs from 100–10 reads; 2 miRNAs from 9–1 reads) in fresh maize was analyzed using a qRT-PCR approach (Supplementary Table S2), which showed a significant positive correlation with the quantitative analysis by small RNA-seq (Pearson’s *r* = 0.89, *P* = 7.19 × 10^−7^), demonstrating the high quality and reliability of our small RNA-seq data (Supplementary Fig. S1).

**Figure 1:**
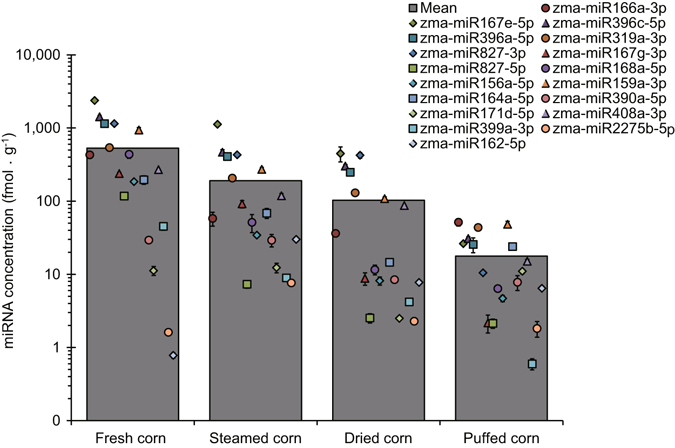
Identification of abundant miRNAs in fresh maize and chow diets. The average plant miRNA concentrations in cooked maize-containing chow diets, including fresh, steamed, dried and puffed maize, were evaluated by q-PCR (*n* = 3).

We next sought to evaluate the concentration of maize miRNAs in cooked maize-containing chow diets (typically steamed, dried and puffed maize). Similar to previous studies on rice and honeysuckle^18, 24^, the plant miRNAs were resistant to the harsh cooking conditions to a certain extent. All 18 maize miRNAs were detected in diverse maize-containing chow diets, even after the harsh puffing treatment (i.e. high temperature and pressure, and apparent starch dextrinization and protein denaturation)^35, 36^, although their concentrations were decreased to one-thirtieth compared with those in fresh maize (Fig. 1).

### Maize miRNAs are present in porcine nonsolid (blood) and solid tissues

**Figure 2:**
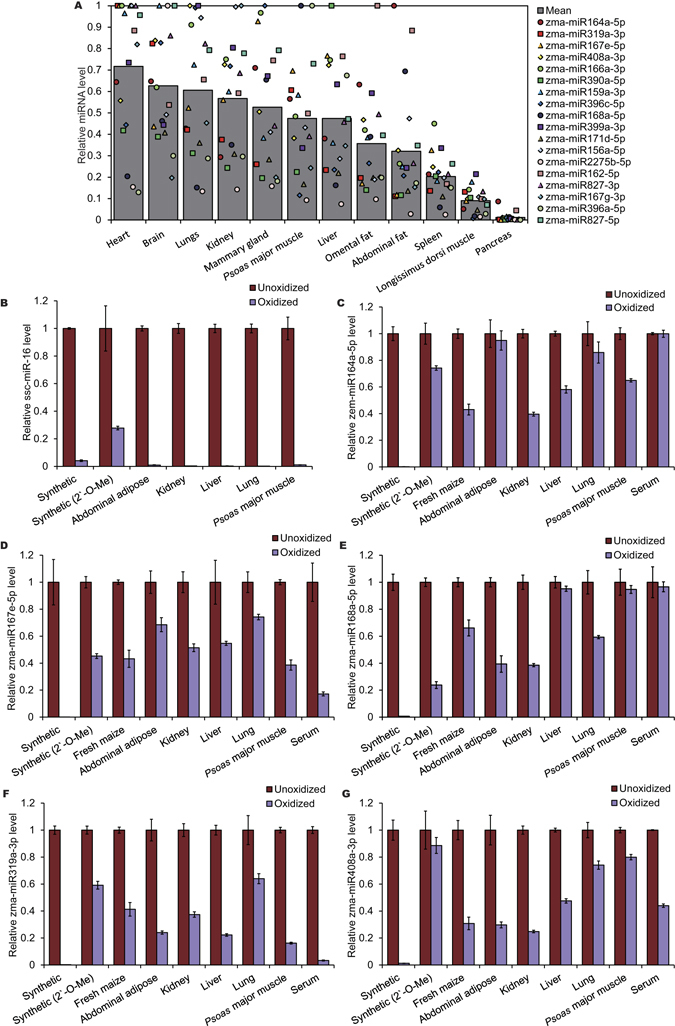
Maize miRNAs are present in porcine nonsolid (blood) and solid tissues. (**A**) The relative expression levels (miRNA/18S, 5S, U6) of 18 plant miRNAs in 12 porcine tissues were detected by qRT-PCR (*n* = 3). Data were normalized to the levels of porcine 18S, 5S and U6, and then plotted relative to the highest levels in the tissues. (**B–G**) Equal amounts of synthetic plant and porcine small RNAs (with or without 2′-O-methylated 3′ ends) and total small RNAs isolated from fresh maize, porcine serum and tissues were treated with/without sodium periodate. After the reactions, the endogenous (**B**) and plant (**C–G**) miRNA levels were detected by qRT-PCR assay. The pigs were fed with fresh maize for 7 days. Data were normalized to the miRNA levels of unoxidized samples (*n* = 3).

To assess the survival of exogenous maize miRNAs in pigs, we measured the relative expression levels of 18 maize miRNAs in nonsolid (blood) and solid tissues of three adult female pigs, which were given fresh maize feed and water *ad libitum* for 7 days, by qRT-PCR. As shown in Fig. 2A and Supplementary Fig. S2A, 16 of the 18 selected maize miRNAs were detected in serum and solid tissues, and exhibited relatively low abundance in pancreatic and longissimus dorsi muscle tissues (Fig. 2A). The terminal nucleotide of plant miRNAs has a 2′-O-methyl modification to resist periodate oxidation, but mammalian miRNAs with free 2′ and 3′ hydroxyls are sensitive to periodate^37^. Hence, oxidization of the nucleotides by periodate can determine whether the plant miRNAs identified in pig were genuine plant miRNAs. We thus treated the total small RNAs isolated from serum and solid tissues with sodium periodate (an oxidizing agent). Consequently, while the endogenous porcine miRNAs (ssc-miR-16, ssc-miR-24 and ssc-miR-25) were completely degraded (Fig. 2B and Supplementary Fig. S2B,C), the maize-derived miRNAs (zma-miR164a-5p, zma-miR167e-5p, zma-miR168a-5p, zma-miR319a-3p and zma-miR408a-3p) exhibited similar abundance to synthetic miRNA with 2′-O-methylated 3′ ends, implying resistance to periodate oxidation, and thus were *bona fide* plant miRNAs (Fig. 2C–G).

### Assessing the absorption of exogenous miRNA in the intestine using an *ex vivo* everted gut sac

**Figure 3:**
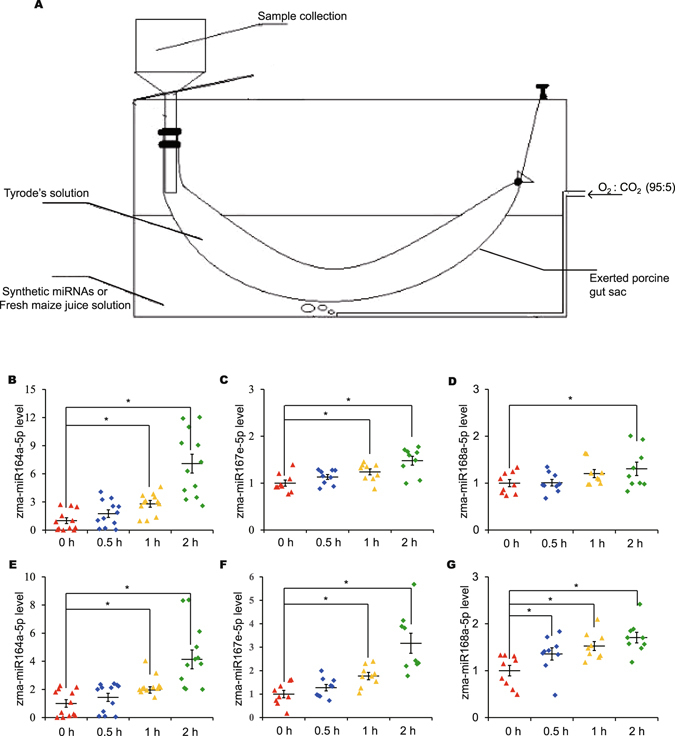
Assessing the absorption of exogenous miRNA in the intestine using an *ex vivo* everted gut sac. (**A**) The everted gut sac method was used to evaluate the absorption of plant miRNAs in the small intestine. An everted porcine gut sac was ligated and placed in synthetic miRNA or fresh maize juice solution, the medium was gassed by bubbling at 37 °C with 95% O_2_ and 5% CO_2_, and the liquid in the internal capsule was collected at different times. (**B–G**) The levels of plant miRNAs in the collected fluid of the internal capsule after the gut sac had been placed in fresh maize juice (**B–D**) or synthetic miRNA (**E–G**) solution. After 0, 0.5, 1 and 2 h, zma-miR164a-5p (**B, E**) (*n* = 12), zma-miR167e-5p (**C**, **F**) (*n* = 9) and zma-miR168a-5p (**D, G**) (*n* = 9) levels were evaluated by qRT-PCR. Statistical significance was determined by Student’s *t*-test (*P < 0.05).

We next sought to assess the absorption of exogenous miRNA in the small intestine using the everted gut sac method (Fig. 3A and Supplementary Fig. S3) because it is an important prerequisite for the hypothesis that diet-derived plant miRNAs are taken up by the GI epithelial cells and enter the circulation^18, 38^. We observed detectably increased concentrations of miRNAs derived from fresh maize juice and synthetic miRNAs with 2′-O-methyl modification in the internal liquid of the intestine (Fig. 3B–G), which suggested that the exogenous plant miRNAs in food could cross the intestinal barrier, although an *in vivo* test was required to further explore the mechanism by which these exogenous miRNAs subsequently enter the blood and solid tissues.

### Dietetically absorbed maize miRNAs may be packaged into exosomes and present in the circulation

**Figure 4:**
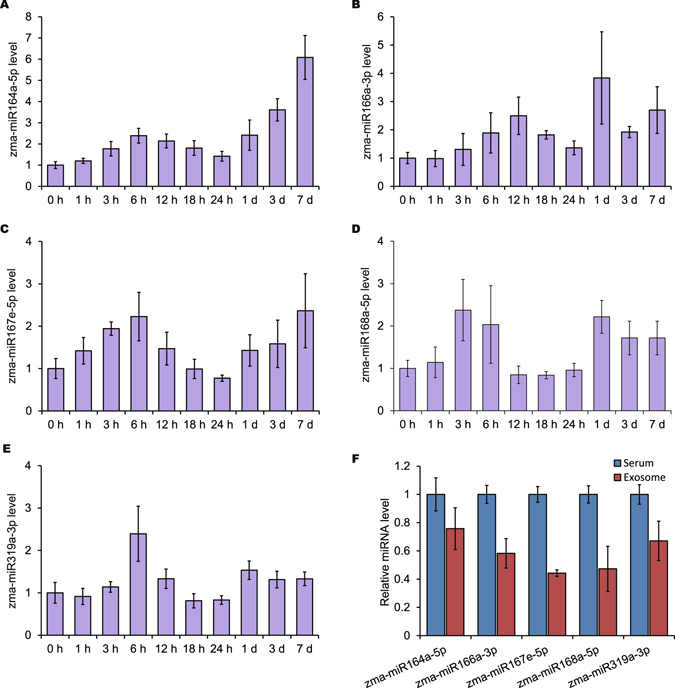
Dietetically absorbed maize miRNAs may be packaged into exosomes and present in the circulation. (**A–E**) The levels of zma-miR164a-5p (**A**), zma-miR166a-3p (**B**), zma-miR167e-5p (**C**), zma-miR168a-5p (**D**) and zma-miR319a-3p (**E**) in porcine serum following one feeding with 1 kg of fresh maize (*n* = 6); after 24 h, the pigs were fed a fresh maize diet for 7 days. The miRNA levels were evaluated by qRT-PCR, and porcine serum was collected after overnight fasting as a control (0 h). (**F**) The levels of plant miRNAs detected by qRT-PCR in exosomes isolated from porcine serum by ultracentrifugation (*n* = 3). Data were normalized to the miRNA levels of serum containing exosomes.

Our findings proved that the exogenous plant miRNAs in food could cross the intestinal barrier *in vitro*. Therefore, we sought to investigate the absorption of plant miRNAs *in vivo* and assessed the levels of maize-derived miRNAs in the serum of pigs fed fresh maize. As shown in Fig. 4A–E, the levels of all five tested maize miRNAs (zma-miR164a-5p, zma-miR166a-3p, zma-miR167e-5p, zma-miR168a-5p and zma-miR319a-3p) gradually increased after one meal of fresh maize, reaching peak values at 6 or 12 hours within the first 24 hours. Following 7 days of access to fresh maize feed *ad libitum*, the maize miRNAs maintained a stable detectable level in the serum (Fig. 4A–E). We next assessed whether these plant miRNAs were located in exosomes in porcine serum by performing ultracentrifugation. All five tested maize miRNAs detected in the serum were primarily present in serum exosomes (~58.2% of the concentration in the serum) (Fig. 4F). Direct PCR-Sanger sequencing confirmed the specificity of our qRT-PCR measurements (Supplementary Table S3)^39^. This result supported the hypothesis that ingested plant miRNAs are taken up by the GI tract and subsequently packaged into exosomes, so as to escape nucleases in cellular compartments and in the bloodstream^18, 25^.

### Possible cross-kingdom regulation of target porcine mRNAs by maize miRNAs

**Figure 5:**
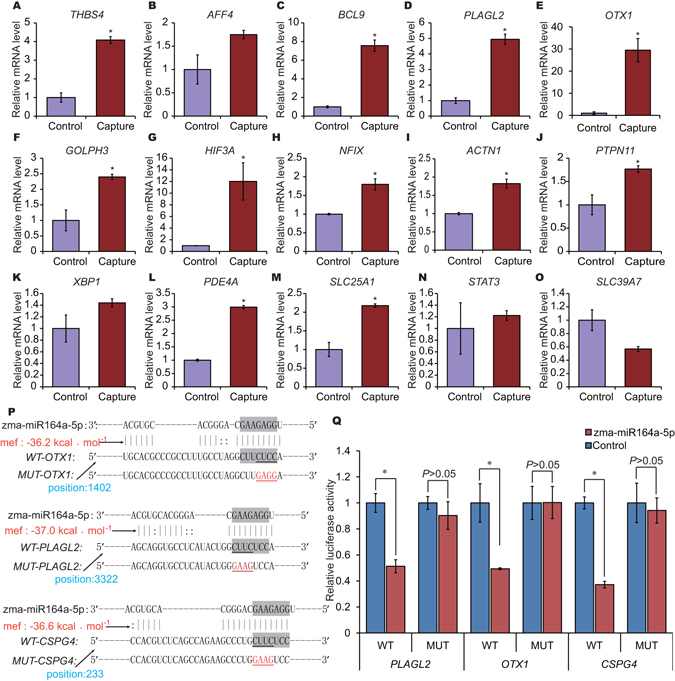
Possible cross-kingdom regulation of target porcine mRNAs by maize miRNAs. (**A–O**) Potential target genes of zma-miR164a-5p were captured in a porcine kidney cell line (PK15) using the MirTrap System. The fold enrichment and statistical significance of the potential target genes were identified by qRT-PCR (*n* = 3). (**P**) Diagram of the putative zma-miR164a-5p binding sites in *OTX1*, *PLAGL2* and *CSPG4*, and luciferase reporter plasmids containing wild-type (WT) or mutant (MUT) putative zma-miR164a-5p target sites. Paired bases are indicated by black vertical lines and mispairing is indicated by two dots. (**Q**) Luciferase activity in the porcine kidney cell line (PK15) co-transfected with zma-miR164a-5p or negative control oligos and the reporter constructs from (**P**) (*n* = 3). Statistical significance was determined by Student’s *t*-test (*P < 0.05).

To confirm earlier findings that exogenous plant miRNAs in food can specifically bind target mammalian mRNAs and influence biological processes^18, 24, 25^, we first performed an *in silico* analysis of the porcine target genes for zma-miR164a-5p, which exhibited a relatively high level in porcine blood and tissues. We predicted 50 potential target porcine mRNAs for zma-miR164a-5p that had a low minimum free energy value (mfe< −25 kcal·mol^−1^) and for which the seed region was highly matched (Supplementary Table S4). Next, the MirTrap System, an RNA-induced silencing complex trap method^40, 41^, was used to isolate specific miRNA target genes in a porcine kidney cell line (PK15). As shown in Fig. 5A–O, of 15 potential zma-miR164a-5p targets analyzed by qRT-PCR, transcripts for 73.33% (11 of 15) were significantly increased, and 53.33% (8 of 15) showed greater than 2-fold enrichment versus the control, which was regarded as a positive result^41^. In addition, we performed a dual-luciferase assay to demonstrate the relationships between zma-miR164a-5p and three of its potential target genes (*CSPG4*, *OTX1 and PLAGL2*) (Fig. 5P). In the three target gene binding sites, zma-miR164a-5p significantly reduced the luciferase activity for wild-type target genes, whereas mutant-type target genes were not affected by transfection with zma-miR164a-5p (Fig. 5Q). These results suggested that dietetically absorbed maize miRNAs are very likely to specifically target endogenous porcine mRNAs and influence gene expression in a fashion similar to mammalian miRNAs.

## Discussion

In the past few years, studies in mammals of circulating miRNAs originating from other species have broadened our view of cross-kingdom gene regulation^42, 43^, but the findings are still controversial because of potential contamination, undetectable abundance and irreproducible results^20, 22, 23^. In the present study, *in vivo* and *in vitro* experiments were performed using diverse and strict methods. After consumption of a fresh maize diet, we found that most maize-derived miRNAs (16 of 18 selected miRNAs) could be detected in porcine tissues, and the plant miRNA concentration in porcine serum was enhanced after feeding on fresh maize. These results are consistent with most previous studies describing that diet-derived plant miRNAs can be detected in other organisms^24–30, 39^.

Many factors including different physiological and pathological conditions could affect the digestion and absorption of food in the GI tract^28, 44, 45^. Yang *et al.* reported that particular diets and/or alterations in intestinal permeability could improve the capacity to absorb small RNAs from the diet^28^. The everted gut sac model is an efficient tool for maintaining normal physiological function of the intestines to study *in vitro* drug absorption and interaction, intestinal metabolism, and the roles of transporters and enzymes^46, 47^. The present experiment using an *ex vivo* everted gut sac supported the assertion that exogenous plant miRNAs can pass through the GI tract. In addition, we further confirmed the authenticity of these plant miRNAs in pigs and the specificity of our qRT-PCR measurements for them by performing a periodate oxidation assay and direct PCR-Sanger sequencing. Taken together, all of our evidence indicated that exogenous plant miRNAs can cross the GI tract and are present in mammals.

Recently, it has been reported that exogenous plant miRNAs can target mammalian mRNAs to regulate the expression of target genes and influence related biological processes^18, 24, 25^. In view of our evidence that dietary plant miRNAs can enter porcine blood and tissues through food intake, a variety of mRNAs might be targeted and affected by exogenous dietary plant miRNAs in different organisms. Interestingly, evidence from the miR-TRAP approach showed that zma-miR164a-5p could exert the same function as endogenous miRNAs in porcine cells and target some of its predicted target genes in the Argonaute/RISC complex. Furthermore, the luciferase reporter assays further confirmed that plant miRNAs could bind to their potential target genes’ binding sites and influence gene expression.

Despite evidence from *in vitro* studies indicating that exogenous plant miRNAs have the potential to regulate host gene expression, further work is needed to determine the levels that they need to reach to exert their effects in other organisms^39^. The current study showed that is invalidation when miRNA expression under a threshold concentration (<100 copies per cell)^48^. Zhou *et al.* reported that a high concentration (>100 copies per cell) of exogenous honeysuckle miR2911 in mouse could inhibit H1N1 to suppress viral infection^24^; nonetheless, most exogenous diet-derived miRNAs may be not reached the threshold concentration in host organisms^20, 21, 30, 39^. However, dietary customs are the long-term process for most organisms, such as herbivores or vegetarians, whose staple foods, such as grains, herbs, fruit and vegetables, are all rich in plant miRNAs^21, 24, 49^. Chin *et al.* reported that long-term oral miR159 ingestion suppressed breast tumor growth^25^, while Sizolwenkosi *et al.* reported that long-term oral administration of three mammalian miRNAs that were 2′-O-methylated like plant miRNAs reduced mouse intestinal tumor burden *in vivo* ^26^. Although these two studies did not clearly indicate the concentrations of these exogenous miRNAs in the mouse body, the long-term effect of dietary miRNAs needs to be considered in further analysis. Considering the potential effect diet-derived miRNAs might have on cross-kingdom communication, more definitive evidence on the mechanisms of absorption and action of exogenous plant miRNAs needs to be provided in future studies, and exciting avenues of plant miRNA-associated physiological and pathological effects could be demonstrated in the future.

## Materials and Methods

### Ethics statement

All research involving animals was conducted according to the guidelines established by the Regulations for the Administration of Affairs Concerning Experimental Animals (Ministry of Science and Technology, China; revised in June 2004) and approved by the Institutional Animal Care and Use Committee of the College of Animal Science and Technology, Sichuan Agricultural University, Sichuan, China, under permit No. DKY-S20143117.

### Small RNA sequencing

Total RNA was extracted from fresh maize using Trizol Reagent (Invitrogen, Carlsbad, CA, USA) according to the manufacturer’s instructions, and small RNAs were purified from PAGE gels. Illumina sequencing of small RNA samples was performed by BGI (Shenzhen, China). After removing the adaptor sequences from the raw data, the clean reads were compared to known mature maize miRNAs from the miRBase database (http://www.mirbase.org/index.shtml) to identify maize miRNAs.

### Maize processing

Fresh maize was bought from a Chinese farmers’ market. It was boiled in water at 100 °C for 20 minutes to obtain steamed maize; meanwhile, fresh maize was treated at 140 °C for 3 hours and 65 °C for 36 hours to obtain dried maize. Puffed maize (HAHNE, Germany) was bought from a Chinese supermarket. All different products of maize were treated with Trizol Reagent (Invitrogen) and isolated small RNA was evaluated by qRT-PCR.

### Animals and diets

Jinhua female pigs were sacrificed after consuming a fresh maize diet for 7 days. Pigs were stunned by electronarcosis; then, porcine blood and tissues were collected immediately after sacrifice, and serum was obtained by centrifugation at 1000 rpm for 15 minutes. In a separate experiment, female pigs were fed one meal with fresh maize (1 kg/pig) after fasting overnight. After a fixed time interval (i.e. 0, 1, 3, 6, 12, 18 and 24 h), serum was collected from a vein in the forearm, from which total RNA was extracted. After 24 hours, pigs were provided with a fresh maize diet *ad libitum*, and serum was collected at 1, 3 and 7 days. Pigs were sacrificed after the last serum collection, after which tissues were collected immediately. All tissues and body fluid samples were frozen in liquid nitrogen and stored at −80 °C until analysis.

### Cells, reagents and oligos

The porcine kidney cell line (PK15) was obtained from the Type Culture Collection of the Chinese Academy of Sciences (Shanghai, China) and cultured in Dulbecco’s modified Eagle’s medium (DMEM; Gibco, Carlsbad, CA, USA), supplemented with 10% fetal bovine serum (FBS; Gibco) and maintained at 37 °C in a humidified atmosphere containing 5% CO_2_. Synthetic miRNA molecules (with or without a 2′-O-methyl-modified terminal nucleotide) were purchased from RiboBio (Guangzhou, China). Stem-loop qRT-PCR primers were obtained from RiboBio and the other primers were purchased from BGI (Supplementary Tables S5 and S6).

### Exosomes purification from serum

Exosomes were isolated from porcine serum by differential centrifugation according to previous studies with slight modifications^50^. Porcine blood was centrifuged at 1000 rpm for 15 minutes in a tabletop centrifuge at 4 °C. The supernatants were collected, diluted with sterile PBS at a 1:1 ratio, and then centrifuged at 1,200 g for 20 minutes, followed by 10,000 g for 30 minutes in a centrifuge at 4 °C to remove cellular debris. The supernatants were then filtered using a 0.22-µm filter (Millipore Corp., Bedford, MA, USA) and centrifuged at 110,000 g for 2 h at 4 °C in an LE-80 ultracentrifuge (Beckman Coulter, Palo Alto, CA, USA), to pellet the exosomes. The supernatant was removed and the pellet was resuspended in PBS (100 µl).

### RNA extraction and qRT-PCR

Total RNA from the serum, exosomes, cells, or tissues was obtained using Trizol Reagent or Trizol LS Reagent (Invitrogen) according to the manufacturer’s instructions, and each sample was eluted in 30 μl of RNase-free water (Takara, Dalian, China). All reverse transcription of miRNA from maize and Jinhua sow samples was performed using the One Step PrimeScript^®^miRNA cDNA Synthesis Kit (Takara) according to manufacturer’s instructions, and qPCR was performed using SsoAdvanced™ SYBR^®^ Green Supermix (Bio-Rad). Stem-loop qRT-PCR was used to evaluate the miRNA level using the Bulge-Loop^TM^ miRNA qRT-PCR Starter Kit (RiboBio) according to manufacturer’s instructions, and the reverse transcription and qPCR primers were also synthesized by RiboBio. Meanwhile, mRNA was reverse-transcribed to cDNA using PrimeScript^®^ 1st Strand cDNA Synthesis Kit (Takara) and qPCR was performed using SYBR^®^ Premix Ex Taq Kit^TM^ II (Takara) and Bio-Rad CFX96TM Real-Time PCR Systems (Bio-Rad, Hercules, CA, USA). All reactions were performed in triplicate, and the absolute or relative expression levels of the target miRNAs and mRNAs were calculated as needed.

### Oxidation of small RNAs with periodate

Total RNA of the fresh maize or porcine tissues and serum was extracted using Trizol Reagent or Trizol LS Reagent (Invitrogen), and synthetic ssc-miR-16, zma-miR164a-5p, zma-miR167e-5p, zma-miR168a-5p, zma-miR319a-3p and zma-miR-408a-3p (with or without 2′-O-methyl) were obtained from RiboBio. Next, 10 μl of total RNA or synthetic miRNA was mixed with 10 μl of NaIO_4_ (0.25 M) and 80 μl of RNase-free water, and incubated at 0 °C for 40 min in the dark. In the unoxidized group, 10 μl of RNase-free water was used to instead of 10 μl of NaIO_4_. Next, the RNA was precipitated, air-dried, dissolved in RNase-free water, and then assayed by stem-loop qRT-PCR via the same procedure as described above.

### The *ex vivo* everted gut sac method

The *ex vivo* everted gut sac method was performed as previously described with slight modifications to assess the absorption of exogenous miRNA in the small intestine^46, 47^. Briefly, the small intestine (10 cm) was collected and flushed through several times with saline solution (0.9% NaCl) at room temperature. The gut was immediately placed in Tyrode’s solution and the medium was gassed by bubbling at 37 °C with 95% O_2_ and 5% CO_2_. As shown in Fig. 3A, the gut was everted gently and ligated at one end, while the other end was connected to a collection tool. Then, 300 pmol synthetic miRNAs (with a 2′-O-methyl-modified terminal nucleotide) or 10% fresh maize juice was dissolved in Tyrode’s solution of external capsule. After the indicated time points (0, 0.5, 1 and 2 h), the fluid in the internal sac (250 µl) was collected to extract total RNA and the miRNA level was evaluated by stem-loop qRT-PCR.

### T-A cloning and Sanger sequencing

Total RNA from porcine serum and tissues was obtained using Trizol Reagent or Trizol LS Reagent. Small RNA was reverse-transcribed to cDNA and stem-loop PCR was performed using Bulge-Loop^TM^ miRNA qRT-PCR Starter Kit (RiboBio); the A-tailing was added using DNA A-Tailing Kit (Takara). Next, the products were cloned into the pMD19-T vector (Takara) and around 40 monoclonals were randomly picked for each miRNA. Then, Sanger sequencing with the M13 primer was used to determine the sequences of the PCR products.

### Mir-Trap System

To capture mRNAs targeted by zma-miR164a-5p, the specific microRNA targets in mammalian cells were identified using the Mir-Trap System kit (Clontech, Tokyo, Japan) according to the manufacturer’s instructions. Briefly, microRNA was co-transfected into porcine kidney cells (PK15) together with pMirTrap Vector using the Xfect™ MicroRNA Transfection Reagent in combination with Xfect Polymer, followed by incubation for 24 h. Then, the transfected cells were lysed using the MirTrap Isolation Kit and the FLAG-tagged RISC complex (including target mRNAs) was immunoprecipitated using the anti-DYKDDDDK beads from the MirTrap Isolation Kit. The bead-bound target mRNAs were then isolated using the NucleoSpin RNA XS Kit. Furthermore, potential target genes of zma-miR164a-5p in pigs were predicted by TargetScan and NCBI Blast, and the minimum free energy value (mef) was evaluated by RNAhybrid. The isolated RNA was analyzed by qRT-PCR to identify the potential target mRNA of zma-miR164a-5p in porcine cells (Supplementary Table S6).

### Dual luciferase reporter assay

Luciferase activity assays involving a dual-luciferase reporter system were performed to evaluate the relationship between zma-miR164a-5p and three of its potential target genes (*CSPG4, OTX1 and PLAGL2*). In brief, the potentially targeted mRNAs containing zma-miR164a-5p binding sites (wild type or mutant type) were synthesized by Tsingke (China). The sequences were cloned into the *SacI* and *XhoI* sites of the pmirGLO plasmid (Promega, USA) at the 3′ end of the firefly luciferase reporter gene. Porcine kidney cells (PK15) were cultured in 24-well plates and when the cells reached about 70% confluence, recombinant pmirGLO vector with wild-type (WT) or mutant (MUT) binding sites was co-transfected with synthetic zma-miR164a-5p or negative control oligos into these cells (Lipofectamine 3000; Invitrogen). Cells were collected after 48 hour, and their dual-luciferase activity was measured using the Dual-Luciferase Reporter Assay System kit (Promega, USA).

### Statistical analysis

Data are presented as mean ± SEM of at least three independent experiments. Statistical analyses were performed using Student’s *t*-tests and the differences were considered significant at *P* <  0.05.

## Electronic supplementary material

Supplementary Information
