# Supplementary material for: Detection of dietetically absorbed maize-derived microRNAs in pigs
Source: Sci Rep. 2017 Apr 5;7:645. doi: 10.1038/s41598-017-00488-y (PMC5428504; doi:10.1038/s41598-017-00488-y)
Supplement: Supplementary file 1 — Supplementary Information [file 41598_2017_488_MOESM1_ESM.pdf]

Supplementary Information for

Detection of dietetically absorbed maize-derived microRNAs in pigs

Yi Luo[1+], Pengjun Wang[1+], Xun Wang[1*+], Yuhao Wang[1], Zhiping Mu[1, 2], Qingzhi Li[1,3], Yuhua Fu[1, 4], Juan Xiao[1], Guojun Li[1], Yao Ma[1], Yiren Gu[5], Long Jin[1], Jideng Ma[1], Qianzi Tang[1], Anan Jiang[1], Xuewei Li[1], Mingzhou Li[1*]

[1]Institute of Animal Genetics and Breeding, College of Animal Science and Technology, Sichuan Agricultural University, Chengdu 611130, China;

[2]Chongqing Three Gorges University, Chongqing 404000, China;

[3] The Fishery Institute of Sichuan Academy of Agricultural Sciences, Chengdu 611731, China;

[4] College of Animal Science and Technology, Huazhong Agricultural University, Wuhan 430070, China;

[5] Animal Breeding and Genetics Key Laboratory of Sichuan Province, Sichuan Animal Science Academy, Chengdu 610066, China;

[+]These authors contributed equally to this work;

*Correspondence should be addressed to Mingzhou Li (email: mingzhou.li@sicau.edu.cn) & Xun Wang (email: xunwang@sicau.edu.cn)

Supplementary Information, Figure S1. Pearson's correlation of miRNA data in fresh maize measured using the qRT-PCR and RNA-seq methods.

Supplementary Information, Figure S2. (**A**) The relative expression levels (miRNA/18S, 5S, U6) of 18 plant and 4 endogenous miRNAs in porcine serum were determined by qRT-PCR (normalized to 18S, 5S, U6; $n = 3$). (**B, C**) Equal amounts of total small RNAs isolated from porcine tissues were treated with/without sodium periodate. After the reactions, the ssc-miR-24 (**B**) and ssc-miR-25 (**C**) miRNA levels were detected by qRT-PCR assay. The pigs were fed with fresh maize for 7 days. Data were normalized to the miRNA levels of unoxidized samples.

Supplementary Information, Figure S3. A barium sulfate assay showed that the everted gut sac was intact. Barium sulfate was added to the external solution. After 0, 0.5, 1 and 2 h, barium sulfate levels in the internal and external solutions were evaluated using OD values.

Supplementary Information, Table S1. Information for miRNA sequencing in fresh maize.

Supplementary Information, Table S2. Sequences of 18 maize-derived miRNAs and 4 endogenous porcine miRNAs.

Supplementary Information, Table S3. TA-cloning and Sanger sequencing results for plant miRNAs identified in porcine serum and tissues.

Supplementary Information, Table S4. List of the *Sus scrofa* potential targets predicted for zma-miR164a-5p.

Supplementary Information, Table S5. Primers for RT-qPCR of endogenous porcine genes.

Supplementary Information, Table S6. Primers for RT-qPCR of potential target genes of zma-miR164a-5p.

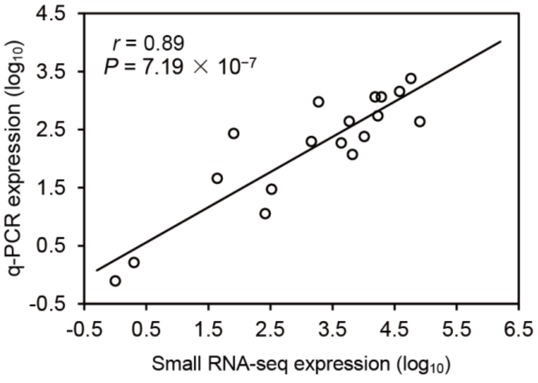

Supplementary Information, Figure S1. Pearson's correlation of miRNA data in fresh maize measured using the qRT-PCR and RNA-seq methods.

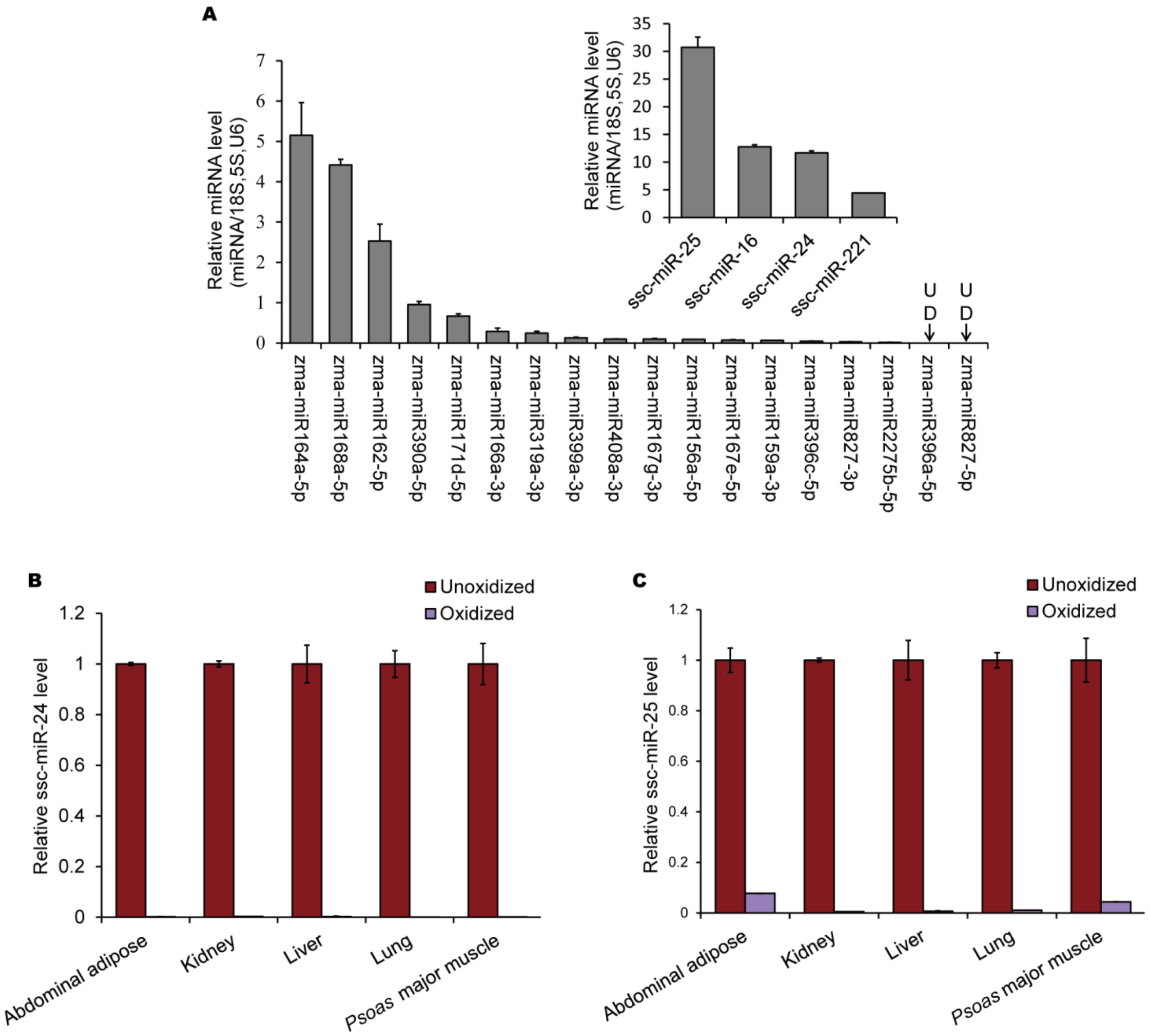

Supplementary Information, Figure S2. (**A**) The relative expression levels (miRNA/18S, 5S, U6) of 18 plant and 4 endogenous miRNAs in porcine serum were determined by qRT-PCR (normalized to 18S, 5S, U6; *n* = 3). (**B, C**) Equal amounts of total small RNAs isolated from porcine tissues were treated with/without sodium periodate. After the reactions, the ssc-miR-24 (**B**) and ssc-miR-25 (**C**) miRNA levels were detected by qRT-PCR assay. The pigs were fed with fresh maize for 7 days. Data were normalized to the miRNA levels of unoxidized samples.

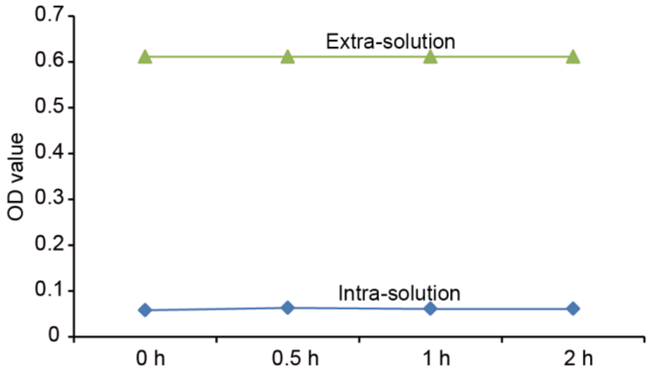

Supplementary Information, Figure S3. A barium sulfate assay showed that the everted gut sac was intact. Barium sulfate was added to the external solution. After 0, 0.5, 1 and 2 h, barium sulfate levels in the internal and external solutions were evaluated using OD values.

Supplementary Information, Table S1. Information for miRNA sequencing in fresh maize.

	MiRNA name	Sequence	Length(nt)	Reads No.
1	zma-miR166a-3p	TCGGACCAGGCTTCATTCCCC	21	80780
2	zma-miR167e-5p	TGAAGCTGCCAGCATGATCTG	21	57916
3	zma-miR396c-5p	TTCCACAGGCTTTCTTGAACTG	22	38043
4	zma-miR396a-5p	TTCCACAGCTTTCTTGAACTG	21	19424
5	zma-miR319a-3p	TTGGACTGAAGGGTGCTCCC	20	17029
6	zma-miR827-3p	TTAGATGACCATCAGCAAACA	21	15360
7	zma-miR167g-3p	GGTCATGCTGTAGTTTCATC	20	10280
8	zma-miR827-5p	TTTGTTGGTGGTCATTTAACC	21	6642
9	zma-miR168a-5p	TCGCTTGGTGCAGATCGGGAC	21	5892
10	zma-miR156a-5p	TGACAGAAGAGAGTGAGCAC	20	4379
11	zma-miR528a-5p	TGGAAGGGGCATGCAGAGGAG	21	4328
12	zma-miR168a-3p	CCCGCCTTGCACCAAGTGAA	20	4112
13	zma-miR159a-3p	TTTGGATTGAAGGGAGCTCTG	21	1884
14	zma-miR164a-5p	TGGAGAAGCAGGGCACGTGCA	21	1448
15	zma-miR166l-3p	TCGGACCAGGCTTCATTCCTC	21	1333
16	zma-miR171b-3p	TGATTGAGCCGTGCCAATATC	21	1079
17	zma-miR167a-5p	TGAAGCTGCCAGCATGATCTA	21	918
18	zma-miR397b-5p	TTGAGCGCAGCGTTGATGAGC	21	648
19	zma-miR166j-3p	TCGGACCAGGCTTCAATCCCT	21	644
20	zma-miR159c-3p	CTTGGATTGAAGGGAGCTCCT	21	601
21	zma-miR394a-5p	TTGGCATTCTGTCCACCTCC	20	594
22	zma-miR393a-3p	ATCAGTGCAATCCCTTTGGAAT	22	569
23	zma-miR166c-5p	GGAATGTTGTCTGGCTCGAGG	21	543
24	zma-miR156l-3p	GCTCACTGCTCTATCTGTCACC	22	388
25	zma-miR396a-3p	GTTCAATAAAGCTGTGGGAAA	21	366
26	zma-miR390a-5p	AAGCTCAGGAGGGATAGCGCC	21	331
27	zma-miR171d-5p	TGTTGGCTCGGCTCACTCAGA	21	261
28	zma-miR397b-3p	TCACCAGCGCTGCACTCAATT	21	234
29	zma-miR395a-3p	GTGAAGTGTTTGGGGGAACTC	21	218
30	zma-miR159i-3p	TTTGGAGTGAAGGGAGTTCTG	21	213
31	zma-miR396e-5p	TTCCACAGCTTTCTTGAACTT	21	163
32	zma-miR399c-5p	GGGTACGTCTCCTTTGGCACA	21	150
33	zma-miR164e-5p	TGGAGAAGCAGGACACGTGAG	21	148
34	zma-miR169o-5p	TAGCCAAGAATGACTTGCCTA	21	146
35	zma-miR167h-3p	GATCATGTTGCAGCTTCAC	19	134
36	zma-miR398a-3p	TGTGTTCTCAGGTCGCCCCCG	21	132
37	zma-miR529-5p	AGAAGAGAGAGAGTACAGCCT	21	128
38	zma-miR167c-3p	GATCATGCTGTGGCAGCCTCACT	23	120
39	zma-miR393a-5p	TCCAAAGGGATCGCATTGATCT	22	103
40	zma-miR160a-5p	TGCCTGGCTCCCTGTATGCCA	21	104

41	zma-miR398b-5p	GGGGCGGACTGGGAACACATG	21	102
42	zma-miR156j-5p	TGACAGAAGAGAGAGAGCACA	21	90
43	zma-miR395e-5p	GTTCCCTTCAAGCACTTCACAT	22	94
44	zma-miR319d-5p	AGAGCGTCCTTCAGTCCACTC	21	81
45	zma-miR408a-3p	CTGCACTGCCTCTTCCCTGGC	21	81
46	zma-miR162-3p	TCGATAAACCTCTGCATCCA	20	77
47	zma-miR166a-5p	GGAATGTTGTCTGGCTCGGGG	21	76
48	zma-miR171f-5p	TGTTGGCATGGCTCAATCAAC	21	70
49	zma-miR398a-5p	GGGGCGAACTGAGAACACATG	21	63
50	zma-miR159e-3p	ATTGGTTTGAAGGGAGCTCCA	21	57
51	zma-miR160f-5p	TGCCTGGCTCCCTGTATGCCG	21	47
52	zma-miR319c-5p	GAGCTCTCTTCAGTCCACTC	20	46
53	zma-miR399a-3p	TGCCAAAGGAGAATTGCCCTG	21	44
54	zma-miR1432-5p	CTCAGGAGAGATGACACCGAC	21	30
55	zma-miR159a-5p	GAGCTCCTATCATTCCAATGA	21	30
56	zma-miR166b-5p	GGAATGTTGTCTGGTTCAAGG	21	29
57	zma-miR164f-5p	TGGAGAAGCAGGGCACGTGCT	21	29
58	zma-miR156i-3p	GCTCACTGCTCTATCTGTCATC	22	28
59	zma-miR171h-3p	GTGAGCCGAACCAATATCACT	21	25
60	zma-miR390a-3p	CGCTATCTATCCTGAGCTCCA	21	24
61	zma-miR156e-3p	GCTCACTGCTCTCTCTGTCATC	22	20
62	zma-miR166g-5p	GGAATGTTGTCTGGTTGGAGA	21	18
63	zma-miR159c-5p	GAGCTCCCTTCGATCCAATCC	21	16
64	zma-miR167j-3p	GATCATGTGGCAGTTTCATT	20	13
65	zma-miR156k-5p	TGACAGAAGAGAGCGAGCAC	20	13
66	zma-miR164g-3p	CACGTGCTCCCCTTCTCCACC	21	13
67	zma-miR172a-3p	AGAATCTTGATGATGCTGCA	20	13
68	zma-miR395b-5p	GTTCCCTACAAGCACTTCACAA	22	12
69	zma-miR395a-5p	GTTCTCCTCAAACCACTTCAGTT	23	12
70	zma-miR164h-5p	TGGAGAAGCAGGGCACGTGTG	21	11
71	zma-miR399e-3p	TGCCAAAGGAGAGTTGCCCTG	21	11
72	zma-miR2118a-3p	TTCCTGATGCCTCTCATTCCTA	22	9
73	zma-miR159b-5p	GTGCTCCCTTCACACCAATAA	21	9
74	zma-miR169m-5p	TAGCCAAGAATGGCTTGCCTA	21	9
75	zma-miR169m-3p	GGCATCCATTCTTGGCTAAG	20	8
76	zma-miR169o-3p	GGCAGGTCTTCTTGGCTAGC	20	8
77	zma-miR171h-5p	TGGTATTGTTTCGGCTCATGT	21	7
78	zma-miR396g-5p	TCCCACAGCTTTATTGAACTG	21	7
79	zma-miR528a-3p	CCTGTGCCTGCCTCTTCCATT	21	7
80	zma-miR167a-3p	GATCATGCATGACAGCCTCATT	22	6
81	zma-miR156d-3p	GCTCACTTCTCTTTCTGTCAGC	22	9
82	zma-miR169h-5p	TAGCCAAGGATGACTTGCCTA	21	5
83	zma-miR166m-5p	GGAATGTTGGCTGGCTCGAGG	21	5
84	zma-miR167e-3p	GATCATGCTGTGCAGTTTCATC	22	5

85	zma-miR166h-5p	GGAATGACGTCCGGTCCGAAC	21	4
86	zma-miR395k-3p	GTGAAGTGTTTGAGGAAACTC	21	4
87	zma-miR169c-3p	GGCAAGTCTGTCCTTGGCTACA	22	4
88	zma-miR159f-5p	GAGCTCCTCTCATTCCAATGA	21	4
89	zma-miR164b-3p	ATGTGCCCATCTTCTCCACC	20	4
90	zma-miR171g-3p	TGAGCCGAGCCAATATCACTT	21	4
91	zma-miR167d-3p	GGTCATGCTGCTGCAGCCTCACT	23	3
92	zma-miR2118d-3p	TTCCTGATGCCTCCCATGCCTA	22	4
93	zma-miR171a-3p	TGATTGAGCCGCGCCAATAT	20	3
94	zma-miR167b-3p	GATCATGCTGTGACAGTTTCACT	23	3
95	zma-miR172b-5p	CAGCACCATCAAGATTCACA	20	3
96	zma-miR171l-5p	TATTGGCGTGCCTCAATCCGA	21	2
97	zma-miR171i-5p	TGTTGGCACGGTTCAATCAAA	21	2
98	zma-miR156b-3p	GCTCACCCTCTATCTGTCAGT	21	2
99	zma-miR2275b-5p	AGGATTAGAGGCAACTGAACC	21	2
100	zma-miR169i-3p	GGCAGTCTCCTTGGCTAG	18	2
101	zma-miR394b-3p	AGGTGGGCATACTGCCAATG	20	2
102	zma-miR166n-5p	GGATTGTTGTCTGGCTCGGTG	21	2
103	zma-miR529-3p	GCTGTACCCTCTCTCTTCTTC	21	2
104	zma-miR171l-3p	GGATTGAGCCGCGTCAATATC	21	2
105	zma-miR399f-3p	TGCCAAAGGAAATTTGCCCCG	21	2
106	zma-miR171g-5p	TATTGACTTGGCTCATCTCTC	21	2
107	zma-miR1432-3p	GGGTGTCATCTCGCCTGAAGCA	22	2
108	zma-miR169a-3p	GGCAAGTTGTTCTTGGCTACA	21	2
109	zma-miR169c-5p	CAGCCAAGGATGACTTGCCGG	21	2
110	zma-miR169r-3p	GGCAAGTTGTCCTTGGCTACA	21	2
111	zma-miR482-3p	CTTCTTCGCGCACCGCCG	18	1
112	zma-miR395c-3p	GTGAAGTGTTTGGAGGAACTC	21	1
113	zma-miR2118e-3p	TTCCTGATGTCTCCCATTCCTA	22	1
114	zma-miR160f-3p	GCGTGCGAGGTGCCAGGCATG	21	1
115	zma-miR2275a-5p	AGAGTTGGAGGAAAGCAAACC	21	1
116	zma-miR2275a-3p	TTTGTTTTCCTCCAATATCTCA	22	1
117	zma-miR172c-5p	CAGCACCACCAAGATTCACA	20	1
118	zma-miR162-5p	GGGCGCAGTGGTTTATCGATC	21	1
119	zma-miR166k-5p	GGATTGTTGTCTGGCTCGGGG	21	1
120	zma-miR169p-5p	TAGCCAAGGATGACTTGCCGG	21	1
121	zma-miR156j-3p	TGCTCTCTGCTCTCACTGTCATC	23	1
122	zma-miR169k-5p	TAGCCAAGGATGACTTGCCTG	21	1
123	zma-miR164d-3p	CACGTGGTCTCCTTCTCCAT	20	1
124	zma-miR164c-3p	CATGTGCCCTTCTTCTCCATC	21	1

Supplementary Information, Table S2. Sequences of 18 maize-derived miRNAs and 4 endogenous porcine miRNAs.

NAME	Squence (5'to3')	Lenth (nt)
zma-miR166a-3p	UCGGACCAGGCUUCAUUCCCC	21
zma-miR167e-5p	UGAAGCUGCCAGCAUGAUCUG	21
zma-miR396c-5p	UUCCACAGGCUUUCUUGAACUG	22
zma-miR396a-5p	UUCCACAGCUUUCUUGAACUG	21
zma-miR319a-3p	UUGGACUGAAGGGUGCUCCC	20
zma-miR827-3p	UUAGAUGACCAUCAGCAAACA	21
zma-miR167g-3p	GGUCAUGCUGUAGUUUCAUC	20
zma-miR827-5p	UUUGUUGGUGGUCAUUUAACC	21
zma-miR168a-5p	UCGCUUGGUGCAGAUCGGGAC	21
zma-miR156a-5p	UGACAGAAGAGAGUGAGCAC	20
zma-miR159a-3p	UUUGGAUUGAAGGGAGCUCUG	21
zma-miR164a-5p	UGGAGAAGCAGGGCACGUGCA	21
zma-miR390a-5p	AAGCUCAGGAGGGAUAGCGCC	21
zma-miR171d-5p	UGUUGGCUCGGCUCACUCAGA	21
zma-miR408a-3p	CUGCACUGCCUCUUCCCUGGC	21
zma-miR399a-3p	UGCCAAAGGAGAAUUGCCCUG	21
zma-miR2275b-5p	AGGAUUAGAGGCAACUGAACC	21
zma-miR162-5p	GGGCGCAGUGGUUUAUCGAUC	21
ssc-miR-16-5p	UAGCAGCACGUAAAUAUUGGCG	22
ssc-miR-24-3p	UGGCUCAGUUCAGCAGGAACAG	22
ssc-miR-25-3p	CAUUGCACUUGUCUCGGUCUGA	22
ssc-miR-221-5p	ACCUGGCAUACAAUGUAGAUUUCUGU	26

Supplementary Information, Table S3. TA-cloning and Sanger sequencing results for plant miRNAs identified in porcine serum and tissues.

	**Serum**		**Tissues**	
	Correct/Total clones	Rate	Correct/Total clones	Rate
zma-miR164a-5p	12/43	27.91%	15/43	34.88%
zma-miR166a-3p	18/42	42.86%	13/39	33.33%
zma-miR167e-5p	16/42	38.10%	20/36	55.56%
zma-miR168a-5p	20/48	41.67%	18/39	46.15%
zma-miR319a-3p	16/43	37.21%	19/35	54.29%

Supplementary Information, Table S4. List of the *Sus scrofa* potential targets predicted for zma-miR164a-5p.

miRNA	Target NCBI Accession	Name	Position	mfe (kcal·mol^{-1})
zma-miR164a-5p	NM_001243061.1	*ACTN1*	2877	-30.2
zma-miR164a-5p	XM_003123929.4	*AFF4*	2102	-33.1
zma-miR164a-5p	XM_005653072.1	*BCL-9*	5255	-29.2
zma-miR164a-5p	XM_005667820.1	*C9H1orf21*	971	-31.9
zma-miR164a-5p	NM_001243672.1	*DBNDD2*	996	-31.7
zma-miR164a-5p	XM_005668786.1	*ETV4*	2013	-29.9
zma-miR164a-5p	XM_005654472.1	*GABRB3*	116	-29
zma-miR164a-5p	XM_005672402.1	*GOLPH3*	384	-26.2
zma-miR164a-5p	XM_005668668.1	*HELZ*	6725	-31.7
zma-miR164a-5p	XM_003355983.3	*HIF3A*	527	-32
zma-miR164a-5p	XM_005653793.1	*HNRNPU*	2491	-28.7
zma-miR164a-5p	XM_003134614.2	*JHDM1D*	4863	-29.8
zma-miR164a-5p	XM_005661609.1	*MEGF10*	701	-29.1
zma-miR164a-5p	XM_003134022.3	*MRPS36*	584	-27.5
zma-miR164a-5p	XM_005652884.1	*NFIX*	1425	-33.3
zma-miR164a-5p	XM_003125096.2	*OTX1*	1402	-36.2
zma-miR164a-5p	XM_005654824.1	*PDE4A*	3274	-32.3
zma-miR164a-5p	XM_005657213.1	*PIWIL2*	641	-28.5
zma-miR164a-5p	XM_003134392.4	*PLAGL2*	3322	-37
zma-miR164a-5p	NM_214054.1	*PLAT*	1451	-28.2
zma-miR164a-5p	XM_003125179.3	*PPM1B*	1104	-30.8
zma-miR164a-5p	XM_005664147.1	*PRICKLE1*	68	-32.1
zma-miR164a-5p	XM_005657333.1	*PTPN11*	3416	-26.7
zma-miR164a-5p	XM_005660116.1	*RANBP6*	2341	-27.9
zma-miR164a-5p	XM_005652670.1	*SLC2A12*	1473	-28.7
zma-miR164a-5p	NM_001190189.1	*SLC25A1*	1440	-29.9
zma-miR164a-5p	NM_001131045.1	*SLC39A7*	2128	-29.1
zma-miR164a-5p	XM_003481802.2	*SNTB2*	757	-29
zma-miR164a-5p	XM_005667816.1	*SMG7*	3461	-28.3
zma-miR164a-5p	XM_005674526.1	*SRD5A3*	398	-28.3
zma-miR164a-5p	NM_001044580.1	*STAT3*	1794	-26.2
zma-miR164a-5p	XM_005658866.1	*TNRC6B*	701	-28.6
zma-miR164a-5p	XM_001929118.4	*TOX4*	2079	-30.9
zma-miR164a-5p	XM_005665203.1	*TRIM62*	496	-38.3
zma-miR164a-5p	XM_005663555.1	*TSPAN2*	249	-28
zma-miR164a-5p	XM_001929540.3	*VANGL1*	723	-30.4
zma-miR164a-5p	XM_005656070.1	*WDTC1*	53	-34.5
zma-miR164a-5p	NM_001271738.1	*XBP1*	48	-29.9
zma-miR164a-5p	XM_003129653.4	*XRRA1*	1585	-33.9
zma-miR164a-5p	XM_003124536.4	*ZKSCAN2*	3170	-30.1

zma-miR164a-5p	XM_013986875.1	*LOC100736763*	196	-36.6
zma-miR164a-5p	XM_005673371.1	*LOC100738312*	1465	-32.1
zma-miR164a-5p	XM_005673355.1	*GLI3*	4615	-35.7
zma-miR164a-5p	XM_003133813.3	*SNED1*	2901	-34.8
zma-miR164a-5p	XM_005671407.2	*PPRC1*	2466	-36.3
zma-miR164a-5p	XM_003128485.4	*CSPG4*	233	-36.6
zma-miR164a-5p	XM_013999230.1	*LOC100520570*	2383	-33.8
zma-miR164a-5p	XM_003123754.4	*THBS4*	192	-41.6
zma-miR164a-5p	XM_005663804.2	*ELFN2*	767	-33.6
zma-miR164a-5p	XM_013987501.1	*ATP13A1*	70	-36.6

Supplementary Information, Table S5. Primers for RT-qPCR of endogenous porcine genes.

NAME	Forward Primer (5' to 3')	Reverse Primer (5' to 3')
U6	TTATGGGTCCTAGCCTGAC	CACTATTGCGGGTCTGC
5S	GCCCGATCTCGTCTGATCT	AGCCTACAGCACCCGGTATT
18S	TTTCGCTCTGGTCCGTCTTG	TTCGGAACTGAGGCCATGAT

Supplementary Information, Table S6. Primers for RT-qPCR of potential target genes of zma-miR164a-5p.

NAME	Forward Primer (5' to 3')	Reverse Primer (5' to 3')
AFF4	AATGGGCACCAGTCTAAAT	GTACTCCTTGGCATTGTCTTA
BCL9	GAATGGGACTAGCGTTACCTGG	TGGTGGTCTCATTGGGTTGTG
GOLPH3	CGTTCTCGACAAATGGGTG	CCGCTTGGTGGCTAAATC
HIF3A	CAATGCCTGGTGCTCATCTGTG	GCAACCTCCGCGATCCTCT
NFIX	GAACTGGATCTTTATCTGGCTTACTT	TGATGTCCGCATCTCCTTGC
OTX1	CCGCACCAGCTCAGCCCTAT	CGGCACCAGGCTCCTTGTAATC
THBS4	GCTCCAGCTTCTACGTGGTC	TTCCTGGAGTCCTTCCACAG
PLAGL2	TACTGTGCCCAGCGATTTG	GCTCCTGTGAGTGGCTCTTCTT
ACTN1	GCCCGTCCTGATGAGAAA	CACCTTGCAGATGCGATT
PDE4A	TGGTACTACAGCGCCATTAGG	CTCCTCCAGTGTCAGCTCAAAT
PTPN11	AAGAAAGTGCCGCTCATG	GGTCCGAAAGTGGTATTGC
SLC25A1	GGGAACAAGGGCTGAAGGG	AGGCGGTCATAACGAAGAAGC
SLC39A7	CAGGCGATGCGTCTACAAC	CTCAGGCAAGACCGAAACC
STAT3	GAAGGACATCAGCGGTAAGA	AGGTAGACCAGCGGAGACA
XBP1	ATGGATTCTGACGGTGTTGA	GGGAGGCTGGTAAGGAACT
